# Response to exercise‐induced blood pressure elevation is blunted in wrist‐cuff automated oscillometric measurement in healthy young college students

**DOI:** 10.14814/phy2.14570

**Published:** 2020-09-15

**Authors:** Tatsuya Sato, Nobutoshi Ichise, Yoshinori Terashima, Aoi Kato, Hiroya Yamazaki, Shunsuke Jimbo, Noritsugu Tohse

**Affiliations:** ^1^ Department of Cellular Physiology and Signal Transduction Sapporo Medical University School of Medicine Sapporo Japan; ^2^ Department of Cardiovascular, Renal and Metabolic Medicine Sapporo Medical University School of Medicine Sapporo Japan; ^3^ Department of Orthopedic Surgery Sapporo Medical University School of Medicine Sapporo Japan

**Keywords:** blood pressure monitoring, circulatory physiology, exercise physiology, oscillometric method

## Abstract

**Background:**

A wrist‐cuff automated oscillometric device is portable and useful for self‐monitoring of blood pressure (BP) at home and outdoors when an upper arm device is not available. Although the height of the forearm in wrist BP measurement is acknowledged to be the major cause of measurement error, it remains unclear whether exercise affects subsequent wrist BP measurement.

**Methods and Results:**

Ninety‐seven healthy college students (median age of 20 years with an age range of 19 to 36 years, 70.1% males) participated in this study. Care was taken to keep the position of the wrist at a level near the upper arm level in BP measurement. At rest, BP measured by a wrist‐cuff oscillometric device (Omron HEM‐6183) was generally acceptable when it was compared with BP measured by an upper arm oscillometric device (Omron HEM‐7130‐HP) and with BP measured by the auscultatory method using a mercury sphygmomanometer. However, the ratio of systolic BP measured by oscillometric devices just after a two‐step exercise test to that before exercise on the wrist (1.22 ± 0.14) was significantly lower than the ratio on the upper arm (1.27 ± 0.14), and the difference was significantly correlated with exercise‐induced increase in pulse rate (Spearman's ρ = 0.23), suggesting a possible role of autonomic nerve activity in the blunted response to exercise‐induced BP elevation in wrist BP measurement.

**Conclusions:**

The results indicate that the blunted response to exercise‐induced BP elevation should be considered in wrist BP measurement when using a wrist‐cuff oscillometric device.

## Graphical abstract

In this study, we found that the response to exercise‐induced blood pressure elevation was blunted in wrist blood pressure measurement compared with upper arm blood pressure measurement. The difference in the response was significantly correlated with the increase in pulse rate, suggesting a possible role of changes in autonomic nerve activity in the blunted response.

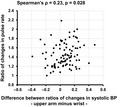

## 1 INTRODUCTION

Noninvasive blood pressure (BP) measurement is important for preventing hypertension‐associated cardiovascular diseases. BP measurement by the auscultatory method using a mercury sphygmomanometer has been the gold standard. However, mercury‐containing products have recently been restricted from the viewpoint of environmental protection, and the use of mercury sphygmomanometers is expected to decrease (Asayama et al., 2016). In addition, the auscultatory method requires training for stable BP measurement and lacks objectivity, leading to inter‐operator differences (Celler, Le, Basilakis, & Ambikairajah, 2017). Furthermore, the auscultatory method is not suitable for self‐monitoring of BP, which is considered to be more important than office BP monitoring to prevent cardiovascular events (Noguchi et al., 2013). The automated oscillometric method, by which measurement of BP is based on pulse waves, not on Korotkoff's sounds, has recently been more popular for BP measurements in various conditions. Although there are still no unified standard measuring protocols, the automated oscillometric method is considered to be an acceptable and alternative method to the auscultatory method for BP measurement.

Major hypertension guidelines recommend the upper arm as the first choice for the site of BP measurement (Uemura et al., 2019; Whelton et al., 2017; Williams et al., 2018). However, a standard cuff cannot be appropriately placed on the upper arm of individuals who are obese or who are wearing thick long‐sleeved clothes. The wrist has been suggested as an alternative site for if the cuff cannot be appropriately placed on the upper arm (Leblanc et al., 2019). Furthermore, wrist BP measurement devices are small, portable, and useful for self‐monitoring (BP) both at home and outdoors. Thus, wrist BP measurement has some advantages, while it is not clear under what conditions wrist BP measurement is not suitable. The height of the forearm in wrist BP measurement is acknowledged to be one of the major causes of measurement error (Casiglia, Tikhonoff, Albertini, & Palatini, 2016); however, it remains unclear whether physical activity affects the accuracy of wrist BP measurement. Notably, it is important to clarify whether BP measurement on the wrist after exercise is as accurate as upper arm BP measurement since wrist‐cuff devices are assumed to be used not only at home but also outdoors. We hypothesized that exercise, which is accompanied by changes in hemodynamics and autonomic nerve function, may affect the accuracy of wrist BP measurement when it is compared with BP measurement on the upper arm.

In the present exploratory study, we examined whether exercise affects BP measurement by a wrist‐cuff automated oscillometric device with comparison to BP measurement on an upper arm in young healthy people. Young subjects were selected for this study because effects of age‐dependent atherosclerosis, which results in impaired physiological pulse wave morphology, on the characteristics of wrist BP measurement needed to be excluded (Smulyan & Safar, 1997).

## 2 MATERIALS AND METHODS

### 2.1 1. Ethical approval

This study was approved by the Ethics Committee of Sapporo Medical University (No. 30‐2‐46) and was strictly conducted in accordance with Ethical Guidelines for Medical and Health Research Involving Human Subjects by the Ministry of Health, Labor and Welfare, Japan. Written informed consent was obtained from all of the participants.

### 2.2 Study design and BP measurement

Of 106 second‐year medical college students who agreed to participate in the study, subjects who had known cardiovascular disease (*n* = 2) and subjects who submitted an incomplete questionnaire about physical information (*n* = 7) were excluded. A total of 97 subjects were enrolled in this study.

In protocol 1, wrist BP at a rest sitting position was measured by a wrist‐cuff oscillometric automated BP monitor (Omron HEM‐6183, Japan) and it was compared with BP measured on the same upper arm by an oscillometric automated device (Omron HEM‐7130‐HP, Japan) and also compared with BP measurement on the upper arm by the classical auscultatory method using a mercury sphygmomanometer (Navis 0610‐B009, Japan). The accuracies of the same oscillometric device and a device similar to those used in this study have been reported by international standard protocols (Saito, Hishiki, & Takahashi, 2019; Takahashi, Yoshika, & Yokoi, 2015). For wrist BP measurement, a cuff of a single (13.5–21.5 cm) attached to the main body of the device (wrist‐cuff device) was used. For upper arm BP measurement, a cuff of a regular size (22.0–32.0 cm) was used. The reason why we used only one size of cuff for each BP measuring site is that there was no obese individual in the participants and that most of the upper arm and wrist circumferences were within the range of indication (Table 1). In each method, BP measurement in the same arm was performed twice with an interval of one minute after sitting for at least 15 min in a chair with a backrest, and the average was used as the value of BP. To prevent the possibility of a biased order of BP measurements affecting results, all of the subjects were evenly divided into three groups and the order of BP measurement by each method was equally allocated. For BP measurements by the oscillometric method, the subjects were instructed how to place the cuff appropriately, and measurements were performed by the subjects pressing the start button according to the instruction manual. The position of the wrist was kept at a level near the upper arm level since it is recognized that the wrist position strongly affects the value of wrist BP measurement (Casiglia et al., 2016). The auscultatory method was performed by a single well‐trained operator.

**Table 1:** The baseline characteristics and BP measurement at rest.

	*N* = 97
Age (years)	20 (20–22)
Men (*N* (%))	68 (70.1%)
Height (cm)	168.5 (162.0– 174.0)
Body weight (kg)	60.0 (52.0–66.8)
Body mass index (kg/m^2^)	20.8 (19.6–22.1)
Right upper‐arm circumference (cm)	23.5 (22.1–25.0)
Right wrist circumference (cm)	15.0 (14.5–16.0)
Pulse rate (bpm)	74 (68–83)
Systolic BP (mmHg)	
Oscillometric method ‐ wrist ‐	109 (100–118)
Oscillometric method ‐ upper arm ‐	108 (101–113)
Auscultatory method ‐ upper arm ‐	109 (104–116)
Diastolic BP (mmHg)	
Oscillometric method ‐ wrist ‐	66 (61–71)
Oscillometric method ‐ upper arm ‐	65 (59–72)
Auscultatory method ‐ upper arm ‐	68 (64–71)

Note

Values are expressed as medians and IQRs (interquartile ranges).

Abbreviation: BP, blood pressure; bpm, beat per minute.

In protocol 2, exercise‐induced changes in BP measured on the wrist were compared to BP measured on the upper arm by the automated oscillometric method. The same oscillometric devices as those used in protocol 1 were used. BP measurements on the wrist and upper arm were simultaneously performed with winding cuffs on separate arms in the sitting position before and after exercise. Master's double two‐step exercise test with slight modification was used for the exercise protocol. In brief, each participant ascended and descended a convex stairway with a height of 9 inches (22.86 cm) at paces of 100 times per minute for 3 min. We did not measure BP during exercise as it has been reported that motion artifacts or noise can affect measurement by the oscillometric method (Shinohara et al., 2017). Instead, BP was measured once and recorded in a sitting position before exercise (i.e., control), immediately after exercise, and after exercise every one minute up to 8 min. BP measurements that showed “error” through the protocol were excluded from analysis. To prevent the possibility of difference in inter‐arm BP values affecting the results, the ratios of BP after exercise to BP before exercise for wrist and upper arm oscillometric measurements on the corresponding arm were compared.

### 2.3 Statistical analysis

Parametric variables were expressed as means ± *SD* and nonparametric variables were expressed as medians (interquartile ranges, IQR). Normality of data distribution was examined by the Shapiro–Wilk test. In protocol 1, comparison of three nonparametric BP values was performed by the Kruskal–Wallis test followed by the Mann–Whitney U post hoc test. Bland–Altman analysis was used to evaluate the agreement among two different BP measurement methods (Bland & Altman, 1986). The 95% limit of agreement (LOA) in nonparametric variables was approximated as the 2.5th percentile and the 97.5th percentile (Shieh, 2018). In protocol 2, comparison of two nonparametric BP values was performed by Wilcoxon's signed‐rank test for nonparametric variables. The ratios of BP just after exercise to that before exercise were normally distributed and were compared by two‐way repeated measures ANOVA followed by the Tukey–Kramer post hoc test. A monotonic relationship between two variables was expressed by Spearman's Rank‐Order Correlation. Differences were considered to be statistically significant if the p‐value was less than 0.05.

## 3 RESULTS

The baseline characteristics of the 97 subjects are shown in Table 1. The median age of the subjects was 20 years with an age range of 19 to 36 years and 70.1% of the subjects were males. The median body mass index (BMI) was 20.8 kg/m^2^ and there were no subjects with obesity defined as BMI of 30.0 kg/m^2^ or higher. The median upper arm circumference was 23.5 cm and the median wrist circumference was 15.0 cm.

In protocol 1, we first compared BP measured on the wrist in a sitting position at rest by the wrist‐cuff automated oscillometric device with BP measured on the upper arm in a sitting position by the automated oscillometric method and with BP measured by the classical auscultatory method. As shown in Table 1, median BPs (IQRs) measured by the wrist oscillometric, upper arm oscillometric, and upper arm auscultatory methods were 109 (100–118), 108 (101–113), and 109 (104–116) mmHg, respectively, for systolic BP and 66 (61–71), 65 (59–72), and 68 (64–71) mmHg, respectively, for diastolic BP. There were no statistically significant differences among the three groups in either systolic BP (*p* = .104) or diastolic BP (*p* = .102).

**Figure 1:**
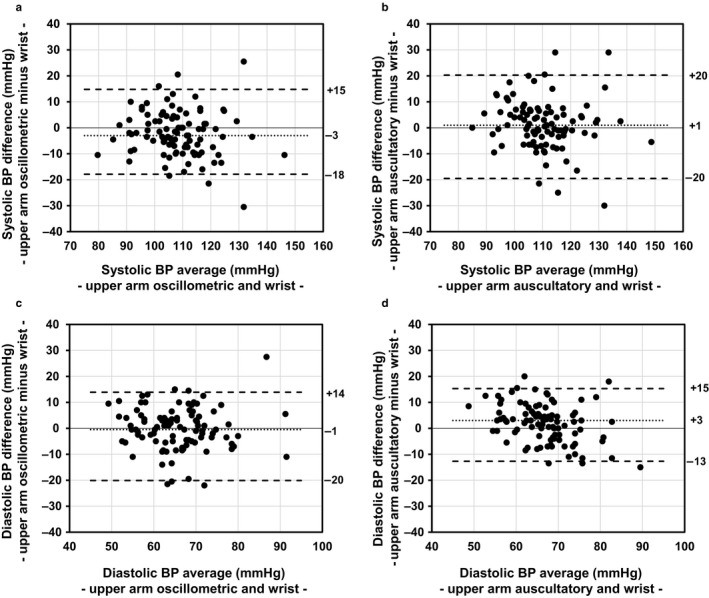
Bland–Altman plots showing the differences between BP values at rest measured by the wrist oscillometric method and upper arm oscillometric or auscultatory method. The X‐axis of each plot represents average values of BP measured by the wrist oscillometric method and upper arm oscillometric or auscultatory method, and the Y‐axis of each plot represents the difference between two methods (upper arm minus wrist). The solid line represents the median difference and the dashed line represents the 2.5th percentile and 97.5th percentile. (a) Difference between systolic BP values measured by the wrist oscillometric method and upper arm oscillometric method. (b) Difference between systolic BP values measured by the wrist oscillometric method and upper arm auscultatory method. (c) Difference between diastolic BP values measured by the wrist oscillometric method and upper arm oscillometric method. (d) Difference between diastolic BP values measured by the wrist oscillometric method and upper arm auscultatory method

We then assessed the validity of wrist BP measurement at rest by comparison with measurements by the upper arm oscillometric and upper arm auscultatory methods using Bland–Altman analysis. As shown in Figure 1, the median differences and 95% limits of agreement (LOA) for wrist BP measurements and upper arm oscillometric BP measurements were −3 (LOA: −18 to +15) mmHg for systolic BP and −1 (LOA: −20 to +14) mmHg for diastolic BP. For wrist BP measurements and upper arm auscultatory BP measurements, the median differences and LOA were + 1 (LOA: −20 to +20) mmHg for systolic BP and +3 (LOA: −13 to +15 mmHg) mmHg for diastolic BP. These results suggest that the agreement between wrist oscillometric BP measurements and upper arm oscillometric BP measurements at rest is generally comparable to the agreement between wrist oscillometric BP measurements and upper arm BP measurements by the classical auscultatory method using a mercury sphygmomanometer.

**Figure 2:**
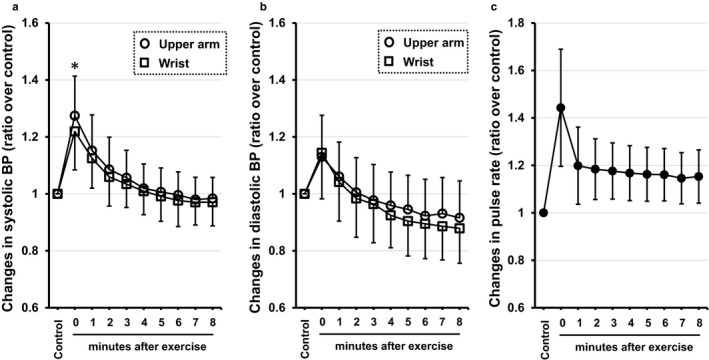
Chronological changes in systolic BP and diastolic BP measured by the wrist and upper arm oscillometric methods and changes in pulse rate before and after the two‐step exercise test. The X‐axis represents time points of the measurements and the Y‐axis represents ratio of BP or pulse rate at the time points from the control value. Values are expressed as means ± *SD*. *p < .05 wrist vs. upper arm. (a) Changes in systolic BP values measured by the wrist oscillometric method (□) and by the upper arm oscillometric method (○). (b) Changes in diastolic BP values measured by the wrist oscillometric method (□) and by the upper arm oscillometric method (○). (c) Changes in pulse rate

In protocol 2, we examined whether exercise affects wrist BP measurement by comparing wrist BP measurement with upper arm BP measurement by the oscillometric method. There was no significant difference between wrist systolic BP and upper arm systolic BP or between wrist diastolic BP and upper arm diastolic BP before exercise (i.e., control) (median systolic BP: 111 [IQR: 105–121] vs. 110 [103–119] mmHg, *p* = .288; median diastolic BP: 72 [66–78] vs. 70 [64–76] mmHg, *p* = .344). Changes in BP after exercise for wrist BP and upper arm BP measurements were expressed as the ratio of change from the control. The ratio of changes in systolic BP just after exercise from the control was significantly lower for wrist BP than for upper arm BP (1.21 ± 0.14 vs. 1.27 ± 0.14, *p* = .006, Figure 2a). The actual value of wrist systolic BP just after exercise was also significantly reduced compared with upper arm systolic BP (135 [IQR: 127–147] vs. 140 [131–150] mmHg, *p* = .010). The tendency for a blunted response of exercise‐induced systolic BP elevation for measurement on the wrist was observed from 1 min after exercise to the end of the protocol (Figure 2a). The difference between the wrist and upper arm in changes in diastolic BP after exercise was not statistically significant (*p* = .144, Figure 2b).

**Figure 3:**
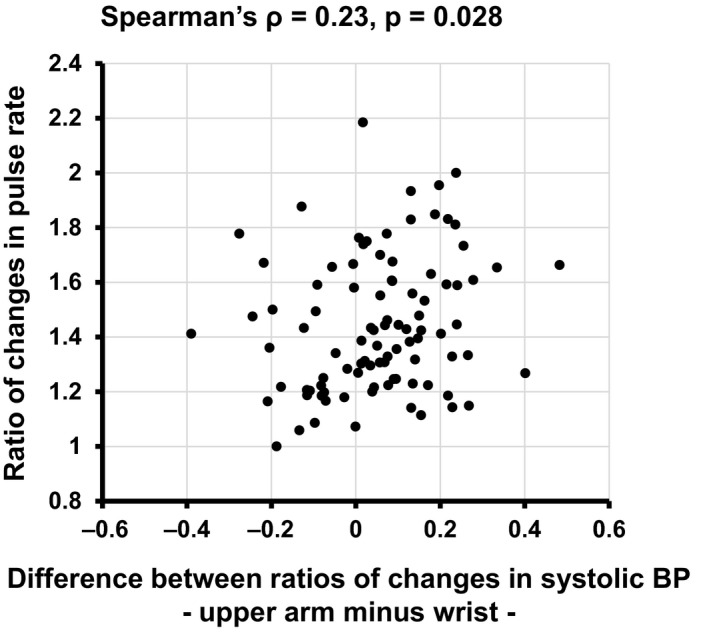
Difference between changes in systolic BP values measured by the wrist and upper arm oscillometric methods just after the two‐step exercise test. The X‐axis of each plot represents the difference between ratios of changes in systolic BP just after exercise from the controls (upper arm minus wrist) and the Y‐axis of each plot represents the ratios of changes in pulse rate just after exercise from the controls. Spearman's correlation coefficient (ρ) and *p*‐value are shown at the top of the figure

Finally, we addressed the possible mechanisms by which the response to exercise‐induced systolic BP elevation just after exercise was blunted in wrist BP measurement compared with that in upper arm BP measurement. Since the autonomic nerve function can regulate vascular resistance, we hypothesized that changes in pulse rate, which is regulated by autonomic nerve activity, are associated with the differences between the wrist and upper arm in the response of BP elevation after exercise. As shown in Figure 3, the difference between ratios of changes in systolic BP just after exercise from controls for upper arm and wrist measurements was weakly, but significantly correlated with the ratio of changes in pulse rate just after exercise from the control (Spearman's ρ = 0.23, *p* = .028), suggesting that exercise‐induced change in autonomic nerve activity is associated with the blunted response of exercise‐induced BP elevation in wrist BP measurements.

## 4 DISCUSSION

This study showed that the response to exercise‐induced systolic BP elevation was blunted in wrist BP measurement compared with that in upper arm BP measurement, whereas resting BP measured at the wrist was similar to that at the upper arm.

It has been demonstrated that blood flow, vascular diameter, and vascular conductance in the radial artery were decreased early after the start of acute dynamic leg exercise in healthy young individuals despite the fact that cardiac output is increased in response to exercise (Elliott, Alsalahi, & Fisher, 2018). Considering that report, increased vascular resistance due to marked vasoconstriction in the radial artery just after the two‐step exercise test may have contributed to the blunted response of exercise‐induced systolic BP elevation at the wrist compared to that at the upper arm in this study (Figure 2a). Even though multiple pathways are thought to be involved in this finding, sympathetic nerve activity has been reported to be one of the predominant regulators of hemodynamic changes in the early stage of exercise (Takahashi et al., 2004; Tsuchimochi, Matsukawa, Komine, & Murata, 2002). Indeed, this study showed that increased pulse rate just after the exercise was associated with the difference between exercise‐induced increase in systolic BP measured at the wrist and upper arm (Figure 3), supporting the rationale that a change in autonomic nerve activity after exercise underlies the mechanism of a blunted response of wrist BP measurement. Furthermore, adrenergic stimulation has been reported to increase the pressure gradient between the proximal femoral artery and distal radial blood pressure via radial artery contraction in patients with septic shock (Kim et al., 2013). The molecular mechanisms underlying the greater sensitivity of the radial artery to vasoconstriction possibly induced by sympathetic nerve activation than that of brachial artery were not addressed in this study. However, considering that the Poiseuille's law, an essential theory of circulating physiology, states that vascular resistance is inversely proportional to the radius to the fourth power, it is possible that the small diameter of the radial artery had a greater effect on vascular resistance induced by vasoconstriction than did the large diameter of the brachial artery.

In addition to exercise, autonomic nerve activity can also be changed by other physiological factors such as circadian rhythm and response to environmental change. Interestingly, Komori et al. reported that systolic BP measured with a cuff‐less wrist ambulatory BP monitoring device was unchanged during sleep but was decreased during the awake period compared to systolic BP measured by a standard arm‐type ambulatory BP monitoring device (Komori, Eguchi, Hoshide, Williams, & Kario, 2013), supporting the notion that changes in autonomic nerve activity can affect wrist BP measurement. Furthermore, a previous study showed that the increase in peripheral BP after cold exposure, which can increase sympathetic tone, was smaller than the increase in central BP (Koutnik et al., 2014). It remains unclear whether or not these physiological factors directly affect wrist BP measurement via alteration of autonomic nerve activity. Nevertheless, it should be considered that changes in autonomic nerve function, which are induced by not only exercise but also other physiological factors, may contribute to the large difference between wrist BP measurement and upper arm BP measurement. Since autonomic nerve function was not able to be evaluated directly in this study, we acknowledge that further study using microneurography, by which autonomic nerve activity can be directly assessed, is needed to investigate the causal relationship between autonomic nerve activity and radial artery conductance after exercise.

In this study, resting BP measured at the wrist and that measured at the upper arm were comparable (Figure 1), suggesting that the bias and the accuracy of wrist measurement were similar to those of upper arm measurement. On the other hand, the anatomical characteristics in BP measurements at the wrist, independently of the accuracy of devices themselves, might have had an effect on the underestimation of BP elevation in measurement at the wrist after the exercise. Since the radial artery is surrounded by hard tendon tissues and distal radius in the wrist, it has been reported that insufficient compression of the radial artery or the degree of wrist extension can affect wrist BP values (Kimura, Chonan, Imai, Goto, & Ishii, 2002). Hence, the possibility that decreased sensitivity in BP detection due to anatomical factors via postexercise radial artery vasoconstriction may have contributed to the blunted response to exercise‐induced BP elevation in the wrist cannot be excluded, although BP measurements showing “error” were excluded from analysis in this study. Nevertheless, the results of this study indicate that underestimation of BP elevation via vasoconstriction in the radial artery, in addition to the position of the wrist, should be considered in wrist BP measurement.

There are a few limitations in this study. First, each BP monitoring method was assessed by only one person and using one type of device. Second, only young healthy people participated in this study. For clinical importance, further studies are needed to determine whether wrist‐cuff BP measurements at rest and after exercise are affected by the presence of risk factors for atherosclerosis such as advanced age, hypertension, diabetes, and renal dysfunction.

In conclusion, the response to exercise‐induced BP elevation was blunted in wrist‐cuff automated oscillometric measurement, and changes in autonomic nerve activity may be associated with the blunted response. The results of this exploratory study suggest that we should consider a state of physical activity when BP is measured by a wrist‐cuff oscillometric device.

## CONFLICT OF INTERESTS

All authors have nothing to declare.

## AUTHORS’ CONTRIBUTIONS

TS and NT conceived and designed the research. TS, NI, and AK organized and performed blood pressure measurements. TS, HY, and NT analyzed data. TS, NI, YT, HY, SJ, and NT interpreted results of experiments. TS and NT prepared figures. TS, NI, YT, and NT drafted and edited the manuscript. All authors read and approved the final manuscript.
